# Cognitive behavioural therapy in practice-nurse led behaviour-intervention for low back pain in German primary care – qualitative process evaluation of a feasibility study

**DOI:** 10.1186/s12912-025-03211-9

**Published:** 2025-05-26

**Authors:** Gregor Feldmeier, Stefan Zutz, Jennifer Höck, Anja Wollny, Peter Kropp, Attila Altiner, Christin Löffler

**Affiliations:** 1 Institute of General Practice, Rostock University Medical Center, Postbox 100888, 18055 Rostock, Germany; 2 Department of General Practice and Health Services Research, University Hospital Heidelberg, Heidelberg, Germany; 3 Institute of Medical Psychology and Medical Sociology, University Medical Center Rostock, Rostock, Germany

**Keywords:** Cognitive behavioural therapy, Chronic back pain, Practice-nurse, Primary care

## Abstract

**Introduction:**

There is evidence that cognitive behavioural therapy (CBT) is effective for patients with chronic low back pain. However, there are by far not enough CBT therapists available to meet this demand. The involvement of specially trained practice nurses could be useful for the implementation of a CBT-based therapy concept in German primary care due to better accessibility and cost-effectiveness. The aim of this pilot study is to evaluate the acceptance of this approach in a pragmatic group programme for patients with chronic back pain.

**Methods:**

In collaboration with a psychologist, psychotherapist and general practitioner, a training concept for chronic pain management, based on behavioural principles was developed. Practice-nurses from one general practice were instructed in performing the training of 5 sessions with a group of up to 6 patients. After completion of the training cycle, acceptability and satisfaction were evaluated through semi-structured interviews with practice-nurses and patients. The content analysis according to Mayring was used for the evaluation. Participants` responses were grouped into general experience, difficulties, stability and security in carrying out of the training and suggestions for improvements.

**Results:**

The pilot study has shown that a practice-nurse based intervention is feasible. A major strength of group training lies in reflecting on the patient’s own problems in comparison with those of the other group members. As a result, patients feel less ill and less restricted in their mobility. The practice-nurses emphasized their positive experiences with the group training, its relevance and usefulness. Nevertheless, they did not believe that it would be sustainable in terms of patient adherence, as they suspected that the majority of patients would not continue such training independently at home.

**Discussion:**

There are few studies describing practice-nurse involvement in the management of patients with chronic back pain based on behavioural principles. To address this gap, we have developed a nurse-led pragmatic group programme. This programme could be an approach for an additional treatment option for affected patients in a multiprofessional general practice. In view of the small sample size and limited Generalisability, further research is required, also to investigate the effectiveness of the approach in routine care in the longer term.

**Trial registration:**

ISRCTN, ISRCTN33541376. Registered 19 February 2024 Retrospectively registered, https//www.isrctn.com/ISRCTN33541376.

**Supplementary Information:**

The online version contains supplementary material available at 10.1186/s12912-025-03211-9.

## Key messages regarding feasibility

**What uncertainties existed with regard to feasibility?:**

It was unclear to what extent the patients would accept the new form of behavioural therapy methods under the guidance of the practice nurse.

**What are the most important results of the feasibility study?:**

The pilot study showed that patients with back pain quickly became involved in the training situation and perceived behavioural therapy measures led by the practice nurse as beneficial. This model is suitable as a complementary approach in general practice and offers the opportunity to address other chronic illnesses, particularly in rural areas with a shortage of GPs and specialists.

**What impact do the results of the feasibility study have on the design of the main study?:**

The qualitative study significantly influenced the design of a subsequent study. Among other things, it became clear which content could be communicated well and which less well. In addition, a revision of the content can better fulfil the wishes of patients, e.g. for relaxation exercises.

**Supplementary Information:**

The online version contains supplementary material available at 10.1186/s12912-025-03211-9.

## Background

### Introduction

In primary care, back pain is the reason for treatment par excellence: In Germany, 85% of all patients are affected at least once in a lifetime [1]. Each year, 25 of women and 17 of men suffer from back pain for longer than three months. The risk of having back pain increases with age and is related to social status: individuals with a low social status (with reference to education, occupation, and income) face a higher risk of back pain than individuals with a high social status [2]. In fact, back pain is the third most common cause of treatment in general practice in Germany, after hypertension and dyslipidemia [3]. The National Disease Management Guideline (NVL) on back pain recommends that avoidable dangerous courses should be ruled out first. If there are no warning signs and there is no indication of nerve compression, a basic diagnosis is sufficient. With regard to therapeutic non-drug measures, the guideline recommends encouraging further exercise, avoiding immobilisation (that means no bed rest) and the application of heat. The use of relaxation techniques such as Jacobson’s Progressive Muscle Relaxation and “training measures (counselling and training) that stimulate and target a return to normal activities” is also recommended [4]. Cognitive behavioural therapy is also mentioned as part of a multimodal treatment concept for chronic back pain. However, behavioural therapy measures have hardly played a role in treating chronic back pain in Germany to date [5, 6]. This is attributable to limited service availability, particularly in rural regions [7]. Moreover, in Germany, behavioural therapy is currently structured predominantly as individual outpatient sessions, while group-based interventions have not yet been systematically implemented [8].

Studies from Great Britain show that behavioural therapy group training can be delegated to practice staff with appropriate training [9, 10]. Given the increasing workload of GPs and psychotherapists and the worsening shortage of GPs, practice-nurses have been gaining in importance in Germany for some years now. In addition to ensuring the specific practice procedures in a GP practice, practice-nurses have an essential task in supporting older and sick people. Practice-nurses therefore also make a significant contribution in interpersonal and social matters. Involving this professional group in the care of patients with chronic back pain could also be a way forward for Germany. Practice-nurses are already increasingly taking on delegable medical services to relieve GPs (e.g. VERAH^®^, NÄPA, EVA or AGnES) [11].

### Behavioural therapy approaches

Research agrees on the benefits of psychosocial interventions and, in particular, behavioural elements in the treatment of chronic pain in general [12] and back pain in particular [13–15]. A systematic review of 22 studies published in 2022 on the effectiveness of cognitive behavioural therapy for chronic low back pain showed that this approach can reduce the perception of pain. The effect is strongest when cognitive behavioural therapy is supplemented with other therapy methods. Furthermore, behavioural therapy can support anxiety avoidance and effectively improve self-efficacy [16].

As early as 2000, Linton published the results of his behavioural therapy programme for preventing the chronification of back pain [17]. Patients from family practices were trained in six behavioural therapy sessions. The control group received a written information sheet. One year after the intervention, the risk of chronification had decreased to 1/9 compared to the control group. At a follow-up survey five years later, participants had significantly less pain, higher levels of activity, better quality of life and better general health compared to the control group. In addition, Linton showed that treatment costs were lower in the intervention group than in the control group [18]. In 2010, Lamb et al. published the results of a randomised controlled trial on the effect of a group programme (BeST, Back Skills Training) based on elements of cognitive behavioural therapy. Participants in the intervention group received 6 weekly small-group training sessions delivered by psychologists, physiotherapists, occupational therapists and practice-nurses. The focus was on gradually increasing activity, on self-management techniques for pain, and on recognizing avoidance behaviour. After 12 months, the pain had decreased significantly and functionality had improved [19, 20]. The BeST protocol adopts a multimodal approach by combining physical activity and behavioral interventions, with a distinctive emphasis on the active involvement of nursing staff. A systematic review by Htay et al. demonstrated that advanced practice nurses contribute to enhanced patient care, increased cost-effectiveness, improved service efficiency, and higher patient satisfaction with overall care quality. Practical implications include a reduction in physician workload and shorter delays in diagnosis and treatment. The review also emphasized the need for future research to address and improve the methodological quality of studies in this field [21]. Based on these approaches, we designed a concept for cognitive behavioural training for patients with chronic back pain. In a pilot study, the concept was taught to practice-nurses and tested with patients in German primary care.

### Training programme, implementation, and evaluation

The training concept was designed interprofessionally by a psychologist, a psychological psychotherapist and a general practitioner. The team also carried out the training of the two practice-nurses who finally implemented the concept in the GP setting. Subsequently, the intervention was carried out in a GP practice in three rounds with a total of 15 patients. During the intervention implementation, regular exchange and mentoring of the practice-nurses by the intervention developers took place. The training programme for the patients consisted of five sessions of 90 min each and included in particular the practice of progressive muscle relaxation according to Jacobson and the teaching of a specific topic in each case. The topics included (1) the influence of mood on pain perception, (2) pain medication and relaxation options, (3) the role of attention in pain perception and coping, (4) pain-related thought and evaluation processes and (5) the prevention of pain exacerbations, including reflection on the content and one’s own goals. The selection of instruments in relation to the content was based on a holistic approach to describing the effects of chronic pain and the existing and validated questionnaires for this purpose. The training programme was evaluated both quantitatively and qualitatively.

For the quantitative evaluation, we used patient-based questionnaires. These measured pain, quality of life, anxiety, depression, and limitations in everyday life. Given the small number of cases, the quantitative evaluation has only an exploratory character. The pain intensity, measured with the Brief Pain Inventory (BPI) [22], was lower at the end of the intervention than at the beginning, increased again within three months after the end of the intervention, but remained below the baseline value. The Short Form Health Survey (SF-12) [23] showed sustained improvement in both mental and physical health over time. Symptoms of depression and anxiety were assessed with the Hospital Anxiety and Depression Scale (HADS) [24]. These values decreased during the intervention period and remained at a lower level three months later. Further outcomes were coping strategies and pain acceptance measured by the Chronic Pain Acceptance Questionnaire (CPAQ) [25]. The latter showed an increase in the total score as a reflection of better coping strategies and increasing pain acceptance. Finally, the Roland and Morris Disability-Questionnaire (RMDQ) [26] was used to measure pain-related disability in daily living. The participants had low disability scores before the training. However, these could be reduced even further at the end of the training. In the following, the article focuses on the question of how the patients perceived the cognitive behavioural therapy led by the practice nurses. In the following, the paper focuses on the qualitative evaluation of the programme.

## Research question

The overarching question of the accompanying qualitative evaluation was how the designed behavioural therapy-oriented group training with psychoeducational elements for patients with chronic back pain carried out by practice-nurses in German primary care was perceived by participating patients. We were particularly interested in the patients’ experiences and evaluations. The evaluation was carried out on the basis of qualitative methods in order to better incorporate patients’ ideas and experiences into the analysis.

## Method

### Study design

For the qualitative evaluation of the pilot study, semi-structured telephone interviews were conducted with eleven patients using an interview guideline. The interviews had an average length of 18 min. In addition, both practices nurses were interviewed by telephone after completion of all training courses. This lasted a total of 180 min. We decided in favour of telephone interviews for feasibility reasons (time and cost restrictions).

### Recruitment of patients

Training participants were recruited in a rural general practice in northern Germany. As part of the pilot study, patient inclusion and exclusion criteria were first established. According to these, patients should be between 18 and 65 years old, have suffered from back pain for at least 6 months and be able to perform the training cognitively and physically. Patients with migraine headaches, severe concomitant illnesses and severe underlying mental illnesses were excluded. Patients taking opiates were only included after direct consultation and consideration with the study team. Using the practice management software, patients with the corresponding ICD codes were identified and recruited based on the inclusion and exclusion criteria. A total of 15 patients participated in the pilot study. Eleven of them agreed to be interviewed. In addition, we interviewed both practice nurses involved.

### Sample description

The patients interviewed were between 35 and 65 years old, the average age was 53 years. We interviewed six women and five men, all of them were German. All participants were married and living in a partnership. On average, the study participants lived with 3 household members. Thus, none of the participants lived alone. Most of the participants had a high-school diploma (Realschulabschluss) (*n* = 8), two participants had a lower school diploma (Hauptschule) and one participant had completed A-levels (Abitur). At the time of the study, six participants were unemployed, one was unable to work and two were pensioners. Two study participants were employed. The study group may be biased: Married patients and unemployed patients were disproportionately represented. The tested intervention could be evaluated differently by unmarried and employed patients. The practices nurses involved were female and aged 24 and 46 respectively. The practice was located in a rural region.

### Data collection

The qualitative study was conducted 12 months after the implementation of the intervention, as the aim was to gain insights into the sustainability of the intervention in addition to the implementation aspects. Each interview was conducted using a specially developed interview guide (Table 1 Interview guide) and began with the following narrative stimulus: “I am interested in how you perceived the group training or pain training and how you have been doing in the meantime. Perhaps you remember that your doctor asked you whether you would like to take part in the training and can tell me about the time from the invitation to the group training programme until today.” The interviewer, a qualified member of staff at the Institute of General Practice, took notes and used narrative-generating conversation prompts to encourage further stories. External follow-up questions followed. These focussed, for example, on possible changes in back pain or willingness to undergo such training again. The practice nurses were asked equivalent questions: “I am very interested in your experiences and impressions during the training. Please tell me about them.” The patients and nurses were interviewed, and no one dropped out of the interview during the course of the study. The patients and nurses were interviewed without the presence of a third person, and no one dropped out of the interview during the course of the study. There was no prior relationship between the interviewer and the patients interviewed. However, the interviewers and practices nurses knew each other from the pilot study. This could have led to distortions due to socially desirable answers. All interviews were recorded, fully transcribed, pseudonymised and analysed.

### Data analyses

The interviews were analysed in a multidisciplinary way by a team consisting of a psychologist (JH), a general practitioner (GF), and two health services researchers (CL, AW). The content analysis according to Mayring was used [27]. In the first step, codes were inductively assigned and then sorted into categories and subcategories. These were then assigned to predefined main categories. This resulted in a total of six main categories: evaluation of the training, participation in training, pain experience, coping with everyday life, use of relaxation exercises, and pain management. Each main category is divided into categories and subcategories. Pain management, for instance, is divided into the categories physiotherapy, acupuncture, medication, and specific therapeutic measures/elements. In total, we coded 94 subcategories. Based on this for each participant group category systems were created by using Microsoft Office. The coding and categorisation system was developed jointly and discussed on an ongoing basis in order to achieve a high level of inter-coder agreement. See appendix for examples. In this publication we summarise the most important results.

## Results

### Overall evaluation of the group training

The patients perceived the implementation of the group training by the practice-nurses as very positive, both in terms of social interaction and in terms of the perceived competences in the implementation. The practice-nurses conducting the training were perceived to be very friendly and responsive to the individual needs of the training participants. There was also sufficient room for exchange among the participants. Especially the exchange within the group was found to be extremely helpful by the participants of the group training: Patients who had already found ways to better deal with their pain shared their experiences. The common experience of back pain united all participants and led to group solidarity. Individual participants, however, had problems in sharing confidential information with the other group therapy participants, precisely because they all lived in the same small town (‘Yes, this group session […] when there are…other people with you, especially as you still know some of them in such a small town […] then it’s always not quite…well…favourable’ (A_1012)). In addition, some group members also found it difficult to get involved in the group work (‘Well, opening up in front of others […] that’s not my thing to be honest’ (A_1012)).

Regarding expectations of the training, most participants wanted “some pain to be taken away or to learn how to deal with it” (A_1011, 49 years, female) in order to “have more fun in life again” (A_1015, 65 years, male). In addition to pain reduction, better posture and lower consumption of medication were also expected.

The content of the training was described by the patients as interesting and relevant. Patients who already knew parts of the training from medical rehabilitation rated them as equivalent in comparison. In particular, the topic “Cope with pain” was perceived positively by the participants. Patients were able to integrate what they had learned into their everyday lives. Nevertheless, some of the patients were disappointed because the training did not include exercises for the back. This was particularly the case for those who had experience in inpatient rehabilitation. (‘I thought, I’ll go there, I can participate in sports’ (A_1024, 54 years, female)). This led to disappointment for some participants, especially at the beginning of the training. (‘That it’s something like back school or something like that…that’s still included. That was actually…yes, what I missed at the beginning’ (A_1012, 51 years, male)).

### Strengthening of individual coping strategies

Participants described pain-influencing factors during the interviews, such as weather-dependent pain that intensifies with intermittent infections (“The worst is always this transitional period when the flu comes or something. Then it is always the worst” (A_1023, 57 years, male)). But also, a connection between pain and occupational activity, partly in connection with psychological tension, was described as a trigger for individual pain states. In particular, prolonged pain had an impact on the psychological state of the participants. Dissatisfaction with one’s own life, which could arise as a result of the pain, was also described (“At some point, you can no longer put it away so well” (A_1025, 51 years, female)).

When asked about their coping strategies and possible changes through the group training, the participants predominantly reported a reappraisal of pain with their pain, whereby it was clear to them that complete freedom from pain cannot always be achieved. They partly succeeded in accepting the pain and a certain degree of unchangeability. The group situation also helped them to classify their own pain differently (“Oh God, the others were even worse, and then somehow … you repress it and … don’t think about it so often” (A_1011, 49 years, female)).

The participants were able to develop personal coping strategies for certain everyday situations and to retain earlier, self-developed coping strategies. According to the participants, this made it possible to avoid overstraining situations or to adapt stress to individual resilience (“Either I leave it out altogether or only do a little, and the next day I do a little more again…and you divide it up better” (A_1005, 57 years, male)).

### Pain perception and pain management

The majority of the participants told us that their pain had been reduced by participating in the group training. While some described a reduction in pain, for others the training led to freedom from pain. Only a few participants described an increase in pain and a general deterioration in their health.

There were also participants who did not experience any change in their pain perception through the group training. They suspected that the short time span of the training compared to the chronic pain that had already existed for a long time was partly responsible for this. This group in particular had often already tried different approaches to reduce pain. Often with little success: “I’ve done all that myself” (A_1023, 57 years, male).

Most of the participants had already used physiotherapy to reduce their pain, but their experiences varied. While some participants found the effect of physiotherapy to be superior to group training (“Then I prefer physio…it relieves more” (A_1025, 51 years, female)), others rated both approaches as equivalent (“It is somewhat similar when you relax with this CD…you also have to do a lot of exercises. Yes…it was actually similar” (A_1011, 49 years, female)). Especially the progressive muscle relaxation according to Jacobson, which was regularly used in the group training, was found to be helpful and pleasant by the participants. After the end of the group training, some patients used the relaxation CD every day. Most patients used the relaxation CD occasionally. But even with occasional use, the positive experience was the decisive motivation (“the feeling was pleasant and calming…when the muscles relax, that’s nice…you feel lighter, that’s right…more relaxed, so to speak” (A_1015, 65 years, male)).

The effects of the group training on the medication taken were assessed differently by the participants. Some patients had to continue taking their painkillers unchanged (“Without tablets it doesn’t work at all. Because I can’t sleep at night” (A_1013, 46 years, female)). Others were able to limit the use of painkillers to “extreme emergencies” (A_1014, 35 years, male).

Especially through the use of relaxation exercises, patients were able to reduce their pain medication (“This muscle relaxation, **l**ed to me having to take less pain medication. That was a good thing” (A_1015, 65 years, male)). Others were able to stop their pain medication altogether (“Well, at least I managed to get to the point where I don’t take pain medication anymore … because of this thing, I would say … this pain training” (A_1012, 51 years, male)).

From the perspective of the practice nurses, the group training was associated with positive experiences and perceived as both relevant and beneficial. However, they expressed doubts regarding the long-term sustainability of the intervention.: “Patients don’t want to understand it, and they also have to actively do something, and that’s where patients reach their limits. It will work well for a few individuals. The majority will not continue such training and behavioural change independently in their homes” (N_02, 46 years, female).

Additionally, the nurses suggested that it would be beneficial to have the opportunity to group the patients—whom they were already familiar with from the GP practice more appropriately based on specific characteristics. “The age difference should not be too great for the composition of the group … the age range was too great. We know our patients and the groups should be mixed with active and passive patients” (N_02, 46 years, female).

## Discussion

### Summary of findings

Overall, the implementation of patient training for chronic back pain by practice nurses in general practices was evaluated positively. The content was considered engaging, although some elements were familiar—particularly to patients with prior rehabilitation experience. Group-based sessions were seen as especially beneficial and, in some cases, rated as equally effective as physiotherapy. Several participants expressed a desire to integrate the group-based cognitive behavioural therapy approach with physiotherapy elements, especially those with a history of inpatient rehabilitation and therefore differing expectations of the programme offered. Some patients reported reduced use of pain medication during the intervention, with one individual discontinuing it entirely. However, the practice nurses raised concerns about the long-term sustainability of the intervention, particularly regarding whether patients would continue practicing the exercises independently. These concerns are mitigated by findings from Riecke et al., who demonstrated that cognitive behavioural therapy can lead to sustained improvements even eight years after treatment [28].

### Findings in the research context

The intervention tested in this study was associated with a reduction in the burden of chronic back pain in some patients, accompanied by a decrease in the use of pain medication. These findings are consistent with previous research demonstrating the benefits of CBT-based group therapy. A novel aspect of our study, in contrast to the BeST protocol by Lamb et al., is the independent delivery of group sessions by trained practice nurses within general practices. This positions practice nurses as key facilitators in settings where CBT access remains limited - particularly in rural areas.

However, as a pilot study, the primary aim was to assess feasibility rather than efficacy; thus, observed effects should be interpreted with caution. Nevertheless, the results demonstrate that the training concept can be effectively implemented by practice nurses in GP practices and is well accepted by patients.

### Strengths and limitations

The pilot study presented here explores a novel interventional approach for managing chronic back pain in primary care, which was subsequently implemented and evaluated using both quantitative and qualitative methods. Due to the limited number of participants, the findings cannot be generalised, and a larger randomised controlled trial would be required to draw robust conclusions about the intervention’s effectiveness. An important suggestion from the nurses was to better group the patients - whom they already knew from the GP practice - themselves based on certain characteristics. Since they knew the patients, they could have better mixed the groups with more active and more passive patients. Additionally, the pre-existing relationship between the interviewer and the participating practice nurses may have introduced bias, potentially leading to socially desirable responses. Despite these limitations, the qualitative evaluation yielded valuable insights into the feasibility, acceptance, and areas for optimisation of the intervention, offering a foundation for its further development.

## Conclusion

### Implications for practice

Our feasibility study demonstrated that patients are receptive to and appreciative of CBT-based group training within the GP practice setting. However, many expressed a desire to integrate practical physiotherapy elements into the programme. Future research could explore these expectations more systematically, for example, through focus group discussions. Currently, such a combination of CBT and physiotherapy is not established in the German healthcare system. Potential pathways for implementation include the targeted training and integration of practice nurses, or the involvement of physiotherapists in primary care—supported by appropriate adaptations of practice infrastructure. This would enable the development of a future oriented, multi-professional GP setting. Successful implementation would require a clear commitment by all stakeholders to acquire relevant expertise in both CBT and physiotherapy. Further studies are needed to design and evaluate a combined intervention tailored for patients with chronic back pain. Such an approach holds particular relevance for improving care quality and ensuring sustainable provision in underserved rural regions.

### Future research

Given the current and anticipated worsening of supply shortages, especially in rural areas of Germany and many other countries, it is essential to enhance care provision within GP practices and expand therapeutic techniques and services for patients with chronic conditions. This would not only improve the appeal of rural living but also increase the attractiveness of the practice nurse role in GP settings. To further develop the approach outlined here, additional studies focusing on evidence generation, health economic evaluations, sustainability assessments, and ongoing evaluation of patient and practice nurse satisfaction are required.

## Electronic supplementary material

Below is the link to the electronic supplementary material.

Supplementary Material 1

## Data Availability

Data is provided within the manuscript or supplementary information files.

## Appendix

**Table:**

Main category	Category	Subcategory
Evaluation of the training	Training content	Interesting and good training
		Familiar training content
		No new knowledge gained
		Internalised training content
		More specific exercises desirable
		Pain management
	Global evaluation	Good implementation by nurse
		As good as rehabilitation
		Pain sensation
	Group experience	Exchange of experience with other people affected
		Good group constellation
		Group setting unfavourable
	Expectation of improvement	Improvement and help expected
		Expected improvement occurred
		Failure of an expected improvement
	Physical training	Other expectations
		Association with back training
		Association with back training failed
	Training materials	Picking up the training materials
		Training materials too extensive
		More illustrative material desirable
Participation in training	Reason for participation	Offer through the family doctor
		Interest in new experiences
		Prospect of pain reduction
	Renewed participation	Renewed participation conceivable
		Ambivalence about renewed participation
		No renewed participation
	Financial participation	Financial participation conceivable
		Weighing up costs and financial situation
		Financial participation not conceivable
	Recommendation	Without justification
		With reason
		No recommendation
	Importance family practice	Decisive
		Not decisive
Pain experience	Increase in pain	Pain increase
		Fear of pain increase
		Severe pain during training
		High pain burden due to other pain triggers
	Reduction of pain	Pain reduction
		Improved well-being
		Short-term improvement
	No change	No change in pain
		No pain reduction through training
	Coping strategies	Altered perception of pain
		Changes in the way pain is dealt with
		Arrangement with pain in everyday life
		Behavioural changes due to previous disc herniation
		Development of personal coping strategies
		Confirmation of previous coping strategies
		Work has a distracting effect
		Externalisation of responsibility
		Comparison with others
	Factors influencing pain/Explanatory concepts	Weather-related pain
		Pain worst during cold season
		Pain is dependent on the form of the day
		Pain occurs due to work
		Pain increases when brooding
	Effects of long-term pain experiences	Pain changes personality
		Psychological burden of pain increases
		Less empathy in social environment
Everyday problems/coping with everyday life	Movement restriction	Physically
		Dependent on help
		Low mood
	Time constraints	Limited time resources due to occupation
		Personal time management
	Sustainability of the training content	Limited transfer to everyday life
		Lack of presence
		Lack of personal success
		Relaxation leads to weight gain
Use of relaxation exercises	Individual relaxation methods	By means of Tens device
		Relaxation exercise with ball and mat
		Massage seat for relaxation
	Application of the learned Postisometric Relaxation (PMR)	Pleasant during the training
		Daily application
		Occasional practice/ application
		No application
Pain management	Physiotherapy	Frequent use
		Physiotherapy is perceived as more helpful than group training
		Both group training and physiotherapy helpful
		Physiotherapy and group training not comparable
	Acupuncture	Helpful method/alternative
		Acupuncture as more intensive help compared to group training
	Medication	Unchanged medication intake
		Taking medication only when needed
		Reduced medication intake
		Discontinuation of medication
	Specific therapeutic measures/elements	Cure and rehabilitation
		Autogenic training
		Yoga
		Weight training
		Orthopaedic treatment
		Massages
		back school

## Abbreviation

GP: General practitioner
