# Supplementary material for: Cognitive behavioural therapy in practice-nurse led behaviour-intervention for low back pain in German primary care – qualitative process evaluation of a feasibility study
Source: BMC Nurs. 2025 May 26;24:593. doi: 10.1186/s12912-025-03211-9 (PMC12107762; doi:10.1186/s12912-025-03211-9)
Supplement: Supplementary file 1 — Supplementary Material 1 [file 12912_2025_3211_MOESM1_ESM.docx]

Tab.1 Interview guide

| **GUIDELINES FOR FINAL INTERVIEW WITH PATIENTS** |
| --- |

| **Narrative impulse** | I am interested in how you perceived the group training or pain training and how you have been doing in the meantime. Perhaps you remember that your doctor asked you if you would like to participate in the training and can tell me about the time from the invitation to the group training until today.  Please take as much time as you need. I won't interrupt you while you tell me about it, and I'll make a few notes that I'll come back to later if needed. |
| --- | --- |
| **Follow-up questions** | - Perhaps you can still remember the expectations you had when you took part in the group training and tell me about them. - - Did you feel that your expectations were met? - - How has your pain changed over time? - - And what did you particularly like about the group training? - - What might you not have liked? - - Is there anything else you would have liked or hoped for? - - Is there anything you missed in the group training? - - I am particularly interested in how you are doing with your pain - today. Can you tell me about it? How have you been dealing with - your pain since then? Are you still doing the exercises (for muscle - relaxation) that you learned back then? If so, how often?   - Are you still doing the exercises (for muscle relaxation) that you  learned back then? If so, how often?   - - If such a training were offered again today, ...   - Would you participate again?   - - Would you recommend it to other patients/people? - - Imagine you had received an invitation to a group training session - in another practice that was just as easy for you to reach as your - GP's practice. How would that be? Would you have taken part?   - Could you see yourself paying a fee if this group training session  cost money? |
